# Progress towards the eradication of Tsetse from the Loos islands, Guinea

**DOI:** 10.1186/1756-3305-4-18

**Published:** 2011-02-10

**Authors:** Moise S Kagbadouno, Mamadou Camara, Jérémy Bouyer, Fabrice Courtin, Mory F Onikoyamou, Chris J Schofield, Philippe Solano

**Affiliations:** 1 Programme National de Lutte contre la THA, Ministère de la Santé, Conakry, Guinée; 2 Cirad, UMR CIRAD-INRA CMAEE, ISRA-LNERV, Service de Parasitologie, Dakar-Hann, Sénégal; 3 IRD, UMR IRD-CIRAD 177, CIRDES Bobo-Dioulasso BP 454, Burkina Faso; 4 Direction de la Santé animale, Ministère de l'élevage, Conakry, Guinée; 5 LSHTM (ITD), London WC1E7HT, UK

## Abstract

**Background:**

The tsetse fly *Glossina palpalis gambiensis *is the main vector of sleeping sickness (Human African Trypanosomiasis - HAT) in West Africa, in particular in littoral Guinea where this disease is currently very active. The Loos islands constitute a small archipelago some 5 km from mainland Guinea, where *G. p. gambiensis *is well known as a nuisance and potential disease vector by inhabitants of the three main islands, Fotoba, Room, and Kassa. The National Control Program against HAT of Guinea has decided to eradicate tsetse in Loos islands in order to sustainably protect humans and economic activities. After baseline data collection, tsetse control began on the islands in 2006. On each of the three islands a specific combination of control methods was implemented according to the entomological situation found.

**Results:**

Starting densities before control operations were 10, 3 and 1 tsetse/trap/day in Kassa, Room and Fotoba respectively, but by July 2010, tsetse were no longer caught in any of the sentinel traps used for monitoring. The reduction rate was faster where several control methods were implemented as a combination (impregnated traps and targets ITT, selective groundspraying, epicutaneous insecticide treatment of pigs, and impregnated fences around pig pens), whereas it was slower when ITT were used as the only control method.

**Conclusions:**

This 100% suppression is a promising step in the eradication process, but *G. p. gambiensis *may still occur at very low, undetectable, densities on the archipelago. Next step will consist in assessing a 0.05 probability of tsetse absence to ascertain a provisional eradication status. Throughout these operations, a key factor has been the involvement of local teams and local communities without whom such results would be impossible to obtain. Work will continue thanks to the partners involved until total eradication of the tsetse on Loos islands can be declared.

## Introduction

The Loos Islands form a small archipelago off the coast of Guinea-Conakry, some 5 Km from the nearest mainland, which are home to some 8000 inhabitants. Tsetse (*Glossina palpalis gambiensis *VanderPlanck, 1949) were abundant on the three inhabited islands, Kassa, Fotoba and Room, representing a considerable nuisance to the local fishing communities and to tourism, as well as affecting pig-breeding which is a major activity on Kassa island. Human cases of African trypanosomiasis (HAT) were reported from all three of the inhabited islands from the 1940s, but have not been reported there in recent years [1]. By contrast, the nearby mainland area - especially the littoral mangrove region - represents one of the currently most active areas for HAT transmission [2-5].

Comparison of tsetse from the Loos islands with conspecifics from the nearby mainland areas indicated a low rate of genetic exchange between the three islands [6] and an apparently high degree of genetic separation between the island populations and those of the mainland [7]. The two uninhabited islands - Corail and Blanche - did not harbour tsetse [1]. This invited the possibility of a programme designed to eradicate the flies from the entire archipelago following an area-wide strategy [8] addressing an apparently isolated target population to avoid the risk of reinvasion. If successful, such a programme would offer sustainable protection to the local communities - both from the tsetse nuisance and potential for human and animal trypanosomiases transmission - and would potentially stimulate additional economic activities (particularly increased pig-rearing and tourism). The programme would also serve as a test-bed for tsetse eradication within the context of the African Union PATTEC initiative [9-11]. In addition, the programme would serve as a training centre for the national control team, and serve to illustrate the feasibility of tsetse eradication in West Africa even under conditions deemed highly suitable for the flies (and in the face of perennial socio-political difficulties).

After baseline data collection (see details in [1]) tsetse control began on the Loos islands in 2006. Here we present results of the first phase leading to a 100% reduction in apparent tsetse densities in the sentinel traps used for monitoring.

## Methods

### Study area

The Loos islands are a small archipelago of five islands totalling some 20 sq. km off the coast of Guinea Conakry, West Africa. The two largest islands, Fotoba and Kassa, are each about 10 Km long but rarely more than 1 Km width, with maximum altitude at 162 masl. The nearest to the mainland (Kassa) is about 4-5 km from the Kaloum peninsula of Conakry. Three of the islands - Kassa, Fotoba, Room - are inhabited, with a total population of about 8000 mainly involved with fishing, agriculture (cassava, palm trees, mangoes) and tourism, although bauxite mining was also important during 1950-70. Pig breeding is also an important secondary economic activity in Kassa, with the pigs generally exported to Conakry. The vegetation of the islands is mainly degraded Guinean savannah on the rocky areas, secondary forests in the fallow areas, and vergers of palms (*Elaeis guineensis) *and mango trees; annual rainfall is around 4000 mm.

### Tsetse Population Baseline data and Monitoring

For baseline data collection and subsequent monitoring of the tsetse population densities, a total of 40 sentinel traps were set up on the 3 islands during 2005-6 (20 in Kassa, 18 in Fotoba, 2 in Room). This number was doubled in Kassa and Room from July 2009. These traps were all of the unbaited Vavoua type, and their GPS coordinates were recorded in order to be sure to place them at the same locations for all surveys. Entomological surveys for monitoring were implemented every 3 months, this agenda being adapted to seasons and/or local conditions. For each monitoring survey, traps were left during 3 consecutive days with daily harvest of the cages and counting of the tsetse.

### Control techniques

According to the results of the baseline data collection, a combination of techniques was chosen that was specific to each island. A sequential strategy was implemented: the control began on Kassa in October 2006, in Room in April 2007, then in Fotoba in November 2007. The underlying control strategy was to deploy all potential control methods that could be made available at relatively low cost - including the unimpregnated Vavoua traps for monitoring population density, similar traps and black/blue/black targets impregnated with deltamethrin, impregnated netting deployed around the pig-pens (traps, targets and netting supplied by Vestergaard-Frandsen), insecticide pour-ons for treatment of the pigs (supplied by Bayer AH), and ultra-low dosage (ULD) fogging with cyfluthrin or deltamethrin in selected thickets (Swingtec Gmbh, HD Hudson Manufacturing Co., and Bayer CS). These techniques were applied in a phased approach, partly because of logistic questions affecting the supply of materials, but also because we wanted to gain an idea of the effects of these techniques alone and in combination.

In Kassa, where the baseline data had shown the highest densities of tsetse (probably due to pig rearing) a combination of four different techniques was implemented: the epicutanous insecticide treatment of pigs (pour-ons) and insecticide impregnated fencing around pig pens, together with Impregnated Traps and Targets (ITT), plus selective ULD fogging of vegetation thickets. In Room and Fotoba, livestock were too few to constitute important hosts for tsetse. In Room, the smallest island, ITT were deployed in April 2007, and followed with some ULD fogging. In Fotoba, only ITT were used to be able to know their specific impact.

Details of each technique are as follows.

**Figure 1:**
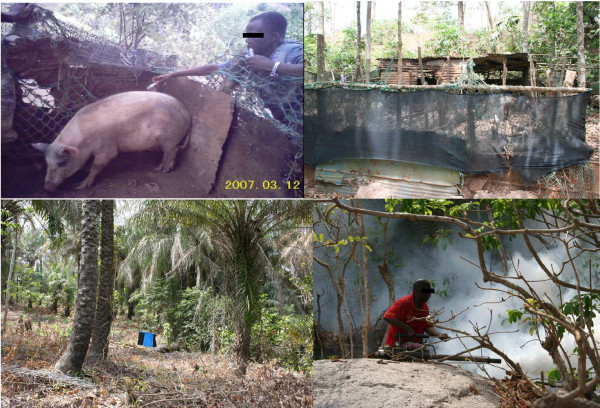
**Pictures illustrating the different control techniques used against *G. palpalis gambiensis *on Kassa island, Guinea**. From upper left to lower right are shown epicutaneous treatment using insecticide (pour on) on pigs, deltamethrin impregnated fences around pig pens, a deltamethrin impregnated black/blue/black target in a palm tree plantation, and cyfluthrin groundspraying using a Swinfog SN50.

**Figure 2:**
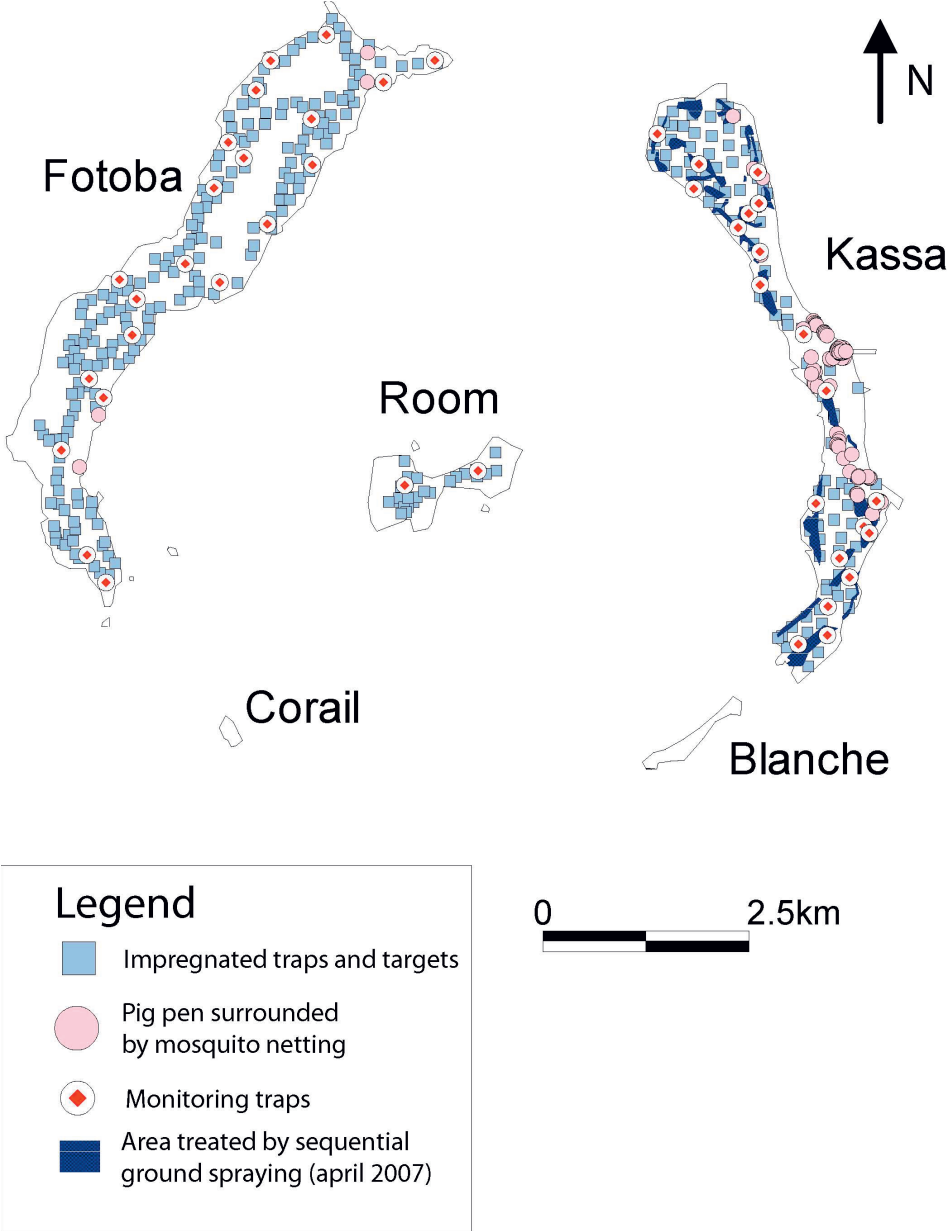
**Combination and GPS location of control techniques applied on Loos islands**. This map shows exact GPS locations of places where the different control techniques (see legend) were implemented on each of the three islands, together with the location of monitoring traps.

- ***impregnated traps and targets***: Vavoua traps [12] and black/blue/black targets [13] factory impregnated with deltamethrin (as supplied by Vestergaard-Frandsen) were deployed as the main control measure. A preliminary spatial analysis had been conducted using a 2002 LANDSAT picture to select suitable sites for tsetse presence, based on the type and density of vegetation, and excluding unfavourable habitats (such as rocky hills). The impregnated traps and targets were initially placed at a density of 30/sq. km, but this was subsequently increased to 60/sq.km. During a preliminary assay in CIRDES, we had measured that the killing effect of the insecticide on these traps (measured as killing at least 50% of the tsetse in contact with the insecticide impregnated cloth) remained for 10 months (Additional file 1). Hence traps and targets were changed every year at the beginning of the dry season (October or November), since it is acknowledged that their efficacy during the rainy season is very poor - mainly due to grass growth reducing their visibility and thus attractiveness. It also has to be noted that tsetse control in the rainy season in Guinea is almost completely impossible due to the heavy rainfall (4000 mm a year, from May to October) which makes access to control sites impossible.

- ***Pour-ons***: After preliminary tests of the toxicity and efficacy of several products and formulations in CIRDES, Burkina Faso (unpublished data), Bayticol pour-on (1% flumethrin) was used 3 times at monthly intervals from oct-nov 2006. It was applied along the dorsal midline of about 1000 pigs each time, using a syringe, at the dose of 1 ml/5 kg of live weight (i.e. 2 mg a.i. per kg) (Figure 1). The Bayticol pour-on tested in similar conditions at CIRDES had a persistency (> 50% of tsetse mortality) of about 20 days (see Additional file 2). The absence of acute toxicity was checked on 104 pigs in farms of Bobo Dioulasso, Burkina Faso. No adverse effects were observed, except moderate pruritus when applied to pigs infested with scabies (data not shown).

- ***impregnated fences***: these consisted of 1 m high netting factory-impregnated with deltamethrin (provided by Vestergaard-Frandsen) that were set around 97 pig pens (Figure 1, and Figure 2). Tsetse visiting these pig pens are knocked down as they touch the fence and then subsequently die [14]. Persistence of the Knock Down effect was estimated on a sample of the netting after 6 months by testing in experimental conditions at CIRDES (see Additional file 2).

- ***ULV fogging***: ground-spraying by ultra-low dose fogging was carried out selectively in areas that appeared to act as tsetse refuges - especially vegetation thickets where the targets were not very efficient. It consisted of 4 interventions every 3 weeks during 2009. For this, cyfluthrin was applied at a nominal rate of 2 g/ha, using two types of machine: 2 Swingfog SN50 thermal foggers (Swingtec GmBH) and one Portapak and one Portastar cold foggers (Hudson Manufacturing). These were shoulder-carried either by two teams of two people walking, or applied from a small boat for costal thickets. Landsat 7 ETM+ images (resolution of 30*30 m) were used to identify pathways for groundspraying that avoided human habitations; these pathways were then recorded as GPS tracks in order to avoid passing more than once or forgetting an area (see Figure 2). Particular attention was paid to wind direction and speed in this insular environment where this is governed by tide, and hours of spraying were organised accordingly.

In addition to these control techniques, we gave special attention to community discussions during each of the interventions. This has been essential to promote community understanding and acceptance of the interventions (and decrease theft and losses of traps and targets) and also to promote active participation of the communities - especially with regard to their knowledge of tsetse refuges, and their help in creating tracks to have access to remote sites, maintenance of fields, regular burning of vegetation, and containment of animals,

## Results

**Figure 3:**
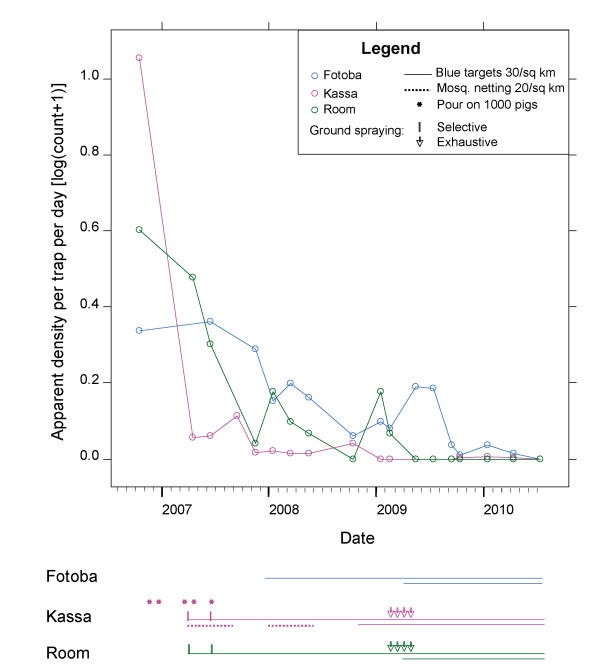
**Overall evolution of *G. p gambiensis *densities according to the different control operations applied on the three islands**. The graph shows the evolution of tsetse densities ([log (count+1)], Y axis) on the three islands according to each monitoring surveys (circles) (X axis showing year and months). Under the graph are detailed the different control methods implemented on each island and the date when they were implemented.

Starting densities in the sentinel traps in October 2006, at the beginning of the dry season and just before the implementation of control measures, averaged 10.33 flies/trap/day (FTD) (range 0 - 102) in Kassa, 3 FTD (range 0 - 6) in Room, and 1.16 FTD (range 0 - 14) in Fotoba. In July 2010, at the beginning of the rainy season, tsetse were no longer caught in any of the sentinel traps of the three islands, indicating an apparent reduction of 100% (Figure 3).

During the implementation of the control measures, tsetse densities varied according to each island and to the control measures implemented, as described below.

### Fotoba

In Fotoba, control operations began in November 2007, with Impregnated Traps and Targets (ITT) at 30/sq.km being the only control measure implemented. Average tsetse density in Nov. 2007, just before the control interventions, was 0.94 FTD. Within the first six months, a reduction of 62% of the initial density was observed (Figure 3), but average fly density then remained stable (around 0.5 FTD) for a year, with only seasonal variations from March 2008 to May 2009 - as if a new population equilibrium had been reached following the initial control pressure. In March 2009, the density of ITT was thus doubled to 60/sq. km, leading to another decrease in average tsetse density during the following months, reaching 0.2 FTD (98% suppression) in October 2009. This suppression level remained constant for several months, but slowly reached 100% in the sentinel traps by July 2010.

### Room

In Room, the control operations began in April 2007 with deployment of 30 ITT/sq.km. This was immediately followed by two selective ground spraying operations of the dry season refuges of tsetse in May and June. The result was a sharp decrease in tsetse densities on this island (97% suppression from the initial density, after 6 months, Figure 3). This tsetse population density then remained fairly constant during the year, with a seasonal peak in January. The suppression then reached 100% for the first time in October 2008, but some tsetse were then caught in January 2009. The density of ITT was thus doubled to 60/sq.km in March 2009 and after 4 successive cycles of groundspraying between February and April 2009, no more tsetse were caught on this island by the sentinel traps during 7 successive monitoring surveys of 3 consecutive days of trapping each.

### Kassa

In Kassa, the initial densities were much higher than in the other islands (~10 FTD in oct. 06, ranging from 0 to 102). The initial reduction in tsetse densities was very fast (98% in 6 months), thanks to a combination of the four control techniques (see Figure 3 for entomological data, and Figure 1 for illustration of the control techniques). Nevertheless, tsetse could still be captured in the sentinel traps, although their densities were very low (in April 07 the average fly density was already 0.15 FTD), mainly in a limited area of the island, in the southern part harbouring dense vegetation. Therefore, four consecutive cycles of groundspraying were implemented in this area in 2009 and the number of ITT was doubled to 60/sq/Km (see Figure 2). Actually, no flies were captured between January and September 2009 (5 consecutive surveys), and only a doubling of the number of sentinel traps allowed to trap again one tsetse in October 2009 at the end of rainy season, with a single fly in January and again in April 2010, showing that *G. p. gambiensis *can survive for a long time at very low densities, below the detection threshold of the original monitoring traps. Since July 2010 however, no further flies have been captured.

## Discussion

This trial was designed to assess the feasibility of tsetse eradication using relatively low-cost methods, in an area of high biological and climatic suitability for *G. palpalis gambiensis*, but with the realistic constraints widespread in the tsetse infested areas of Africa, including low-budget, logistic difficulties, climatic restrictions to access, and perennial uncertainty about the political situation. The word "eradication" here refers to its most recent definition as "the elimination of disease in a defined geographical area as a result of deliberate control efforts" [15], applying it here to tsetse presence rather than disease presence. The trial was prompted by studies of the tsetse populations that indicated negligible apparent gene flow between the mainland and island tsetse populations [7], and low rates of apparent gene flow between the different island populations [6], which suggested that if eradication were achieved on any or all of the infested islands then reinfestation from the mainland would be unlikely [10]. Overall, the results show that a rapid reduction in tsetse abundance was achieved within just a few months following the initial interventions (impregnated traps and targets, together with pour-ons and impregnated nets around pigpens in the case of Kassa island), and that this was perceived to be of great benefit by the local communities due to reduction in the biting nuisance. However, sustained reduction in tsetse abundance required continued intervention, supplemented by additional methods in specific areas.

At this stage, it would appear that tsetse have been eliminated from the islands, in the sense that no flies have been captured on any of the islands, despite intense sampling efforts, since July 2010. However, not catching tsetse in traps does not mean that they are no longer present [16]. Several reasons can account for this: tsetse can be out of the range of the trap attractiveness, especially in thick vegetation, and when unbaited traps are used (lack of attractiveness), or they may be attracted but still not enter the trap (lack of efficacy). It has been recently reported that only ~ 15% of *G. p. gambiensis *attracted to a trap actually enter it [17]. For that reason, for monitoring purposes it may be more appropriate to use attractive devices that do not need capture systems such as cages, which reduce their efficacy. Targets coated with a sticky plastic (to catch tsetse which land on the target) as described by [18], or targets surrounded by electric grids ([19], or see also [17]) might do the job and would be interesting tools to monitor the success of control campaigns. It has also been suggested that tsetse could modify their behaviour during control operations. Amsler et al. [20] suggested that after a control campaign, tsetse were not attracted anymore by the type of traps used during control because of the selection pressure applied, and that there was a need to change the shape of the attractive device to catch the last flies. It has also been suggested that the efficiency of the traps could decrease with the increase of the reduction rate [21], because of density-dependant dispersal [22].

We can apply the model of Barclay & Hargrove [16] to evaluate the probability (or risk) that tsetse were actually present although not captured through a given sampling effort (number of traps*days). In this model, the probability of observing a sequence of zero catches if in fact there are insects in the sampled area is given by:

p=exp (−Stσλ)

where S is the number of traps deployed in the total area, t the number of days for which each trap is operated, σ the trap efficiency and λ the population density (number of insects/area of suitable habitat). For this model, the minimum number of flies in the sample area was set at 2. The trap efficiency, defined as the probability that a trap catches a fly in an area of one square kilometre per day, was defined as 0.01, according to estimates obtained for *G. palpalis gambiensis *by [23]. Applying this model to the tsetse capture data for Room would indicate a requirement for null captures over 19 consecutive sampling occasions in order to achieve a 95% probability that tsetse were indeed absent; for Kassa and Fotoba, this would require null captures over a further 30 consecutive sampling occasions.

Setting insecticide impregnated targets and traps (ITT) at the initial rate of 30/sq km resulted in a marked decrease in density of the targeted tsetse populations, as reported elsewhere for similar ITT densities [24,25], but was not enough to achieve complete suppression. To achieve the apparent 100% reduction in tsetse densities caught in the sentinel traps, the density of ITT had to be increased to 60/sq. km. Then, if supplemented by other techniques such as selective groundspraying, and/or impregnated fences baited with live animals (i.e. pigs in the present case), the suppression was faster than if ITT were used on their own. Other works have reported densities of traps/targets even higher in forest environments where the visibility of the trap is low: up to 250/sq. km were used to control *G. p. palpalis *in Ivory Coast [26]. This is in contrast with areas such as austral Africa where densities of only 4 targets/sq. km [27,28] or even 1 target/sq. km [29] have enabled suppression of tsetse of the morsitans group. It is noteworthy that in this latter area, targets were baited with olfactory attractants (which are currently unavailable for tsetse of the palpalis group) and that the savannah vegetation is more open, allowing a better visibility of targets. Development of efficient olfactory attractants for tsetse of the palpalis group, which now seems feasible [30,17] would greatly assist in the control efforts.

For the Loos Islands, the next step will involve increasing the number of monitoring traps, or changing the traps to more efficient devices that do not need the flies to enter, in order to ascertain the likelihood that flies have been completely eliminated - i.e. when the probability of tsetse presence will be below 0.05 after which control operations will stop. A new monitoring effort would then be implemented 6 months after the end of the control period to allow a potential residual population to regenerate and to declare a definitive "eradication status" in case no further flies are being trapped, as has been done in Zanzibar for *G. austeni *[31]. It is noteworthy that, while waiting for this definitive eradication status for tsetse, disease transmission can already be considered to have been interrupted.

One of the additional results of this trial has also been to show that the total involvement of local teams is essential for the success of any eradication effort. The methods described here have necessarily been continually adapted to local conditions during the project, with the timing of activities adjusted from the original timetable due to various constraints (politico-social events, logistic problems, lack of fuel, and so on). Staff of the National Control Programme (NCP) worked with the local communities to implement the control interventions. They asked for external technical support only when they felt it was necessary. The Guinean team was jointly constituted from the Ministry of Health and the Ministry of Livestock. The people involved were the coordinator (MC), the entomologist of the NCP (MSK), a veterinarian of the Direction of Livestock (OF), and four field assistants in entomology. Locally, additional manpower was recruited when necessary. In addition, there have been several field visits by collaborators based in CIRDES Burkina Faso and IPR Ivory Coast, either as technical support for training, or at progress meetings to discuss strategies, techniques and results.

## Conclusions

The work will continue until total eradication has been obtained from Loos islands, since this seems to be the best guarantee for a sustainable protection of people and their domestic animals against human and animal trypanosomiases. At the same time the NCP will have to face other important challenges, such as implementing vector control in the sleeping sickness foci showing the highest prevalences, such as Dubreka and Boffa. Future projects aiming at eliminating sleeping sickness as a public health constraint will hopefully take advantage of the strengthening capacities of the Guinean team regarding vector control.

## Competing interests

The authors declare that they have no competing interests.

## Authors' contributions

PS, MC, CJS and JB conceived and designed the study, and critically revised the manuscript. MSK was the superviser and main component of all field operations, and drafted the manuscript. FC and MFO have been involved in all the GIS work, analysed the data, and contributed to the manuscript and figures. All authors have approved the final version of this manuscript.

## Supplementary Material

Additional file 1 **Supplementary file 1**. Persistency of the knock-down effect of the blue fabric and mosquito netting used in Loos Island, against laboratory males *G. p. gambiensis *6H after exposure in experimental conditions (95% confidence intervals as vertical bars). Click here for file

Additional file 2 **Supplementary file 2**. Persistency of the knock-down effect of Bayticol 1% Pour on applied on pigs in experimental conditions against laboratory males *G. p. gambiensis *(95% confidence intervals as vertical bars). Click here for file
