# Supplementary material for: Progress towards the eradication of Tsetse from the Loos islands, Guinea
Source: Parasit Vectors. 2011 Feb 10;4:18. doi: 10.1186/1756-3305-4-18 (PMC3048576; doi:10.1186/1756-3305-4-18)

### Supplementary file 1 – Persistency of the knock-down effect of the blue fabric and mosquito netting used in Loos Island, against laboratory males *G. p. gambiensis* 6H after exposure in experimental conditions (95% confidence intervals as vertical bars).


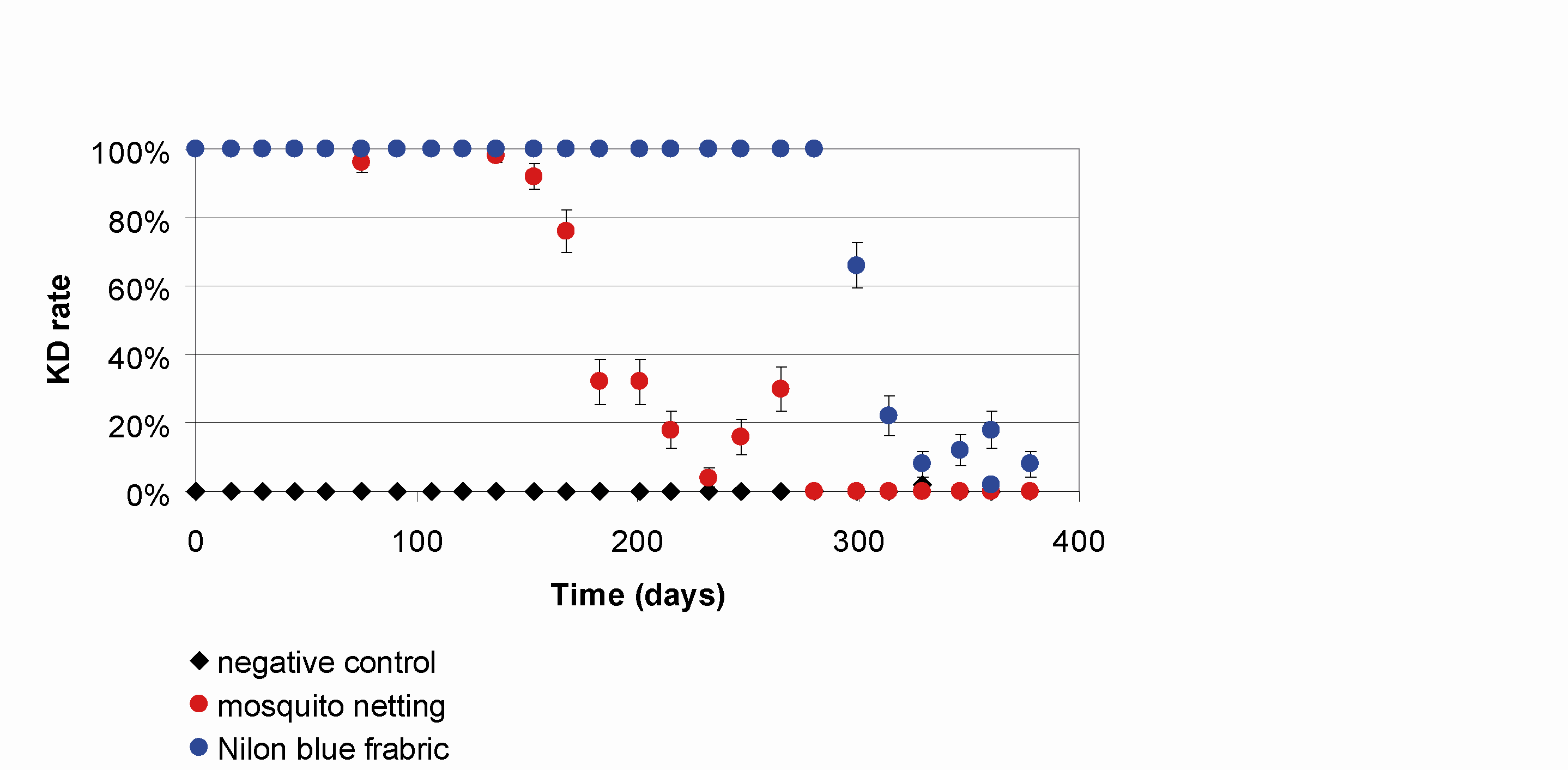

Supplement: Additional file 1 — Supplementary file 1. Persistency of the knock-down effect of the blue fabric and mosquito netting used in Loos Island, against laboratory males G. p. gambiensis 6H after exposure in experimental conditions (95% confidence intervals as vertical bars). [file 1756-3305-4-18-S1.DOC]
