# Supplementary material for: Progress towards the eradication of Tsetse from the Loos islands, Guinea
Source: Parasit Vectors. 2011 Feb 10;4:18. doi: 10.1186/1756-3305-4-18 (PMC3048576; doi:10.1186/1756-3305-4-18)

### Supplementary file 2 – Persistency of the knock-down effect of Bayticol 1% Pour on applied on pigs in experimental conditions against laboratory males *G. p. gambiensis* (95% confidence intervals as vertical bars).


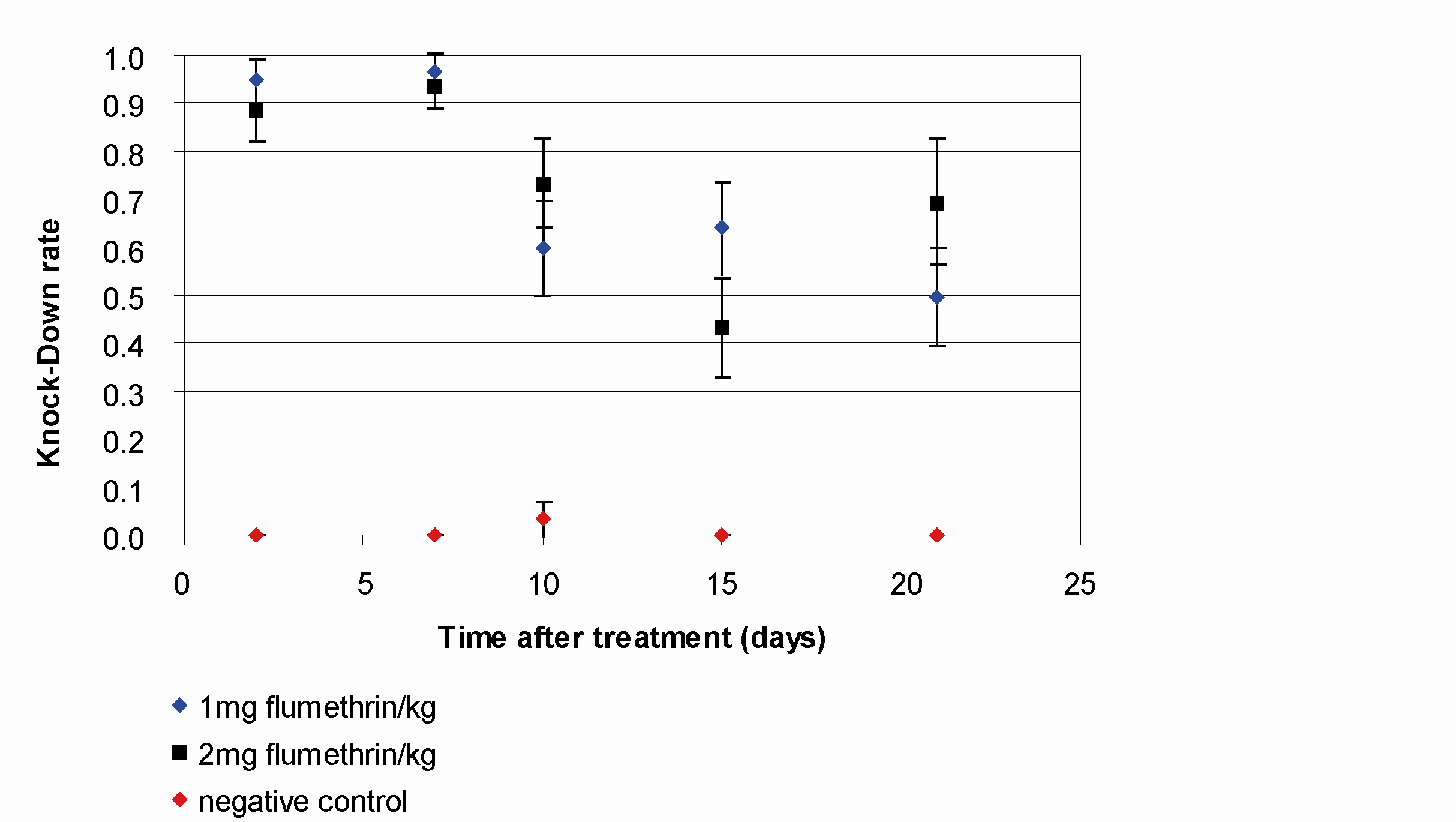

Supplement: Additional file 2 — Supplementary file 2. Persistency of the knock-down effect of Bayticol 1% Pour on applied on pigs in experimental conditions against laboratory males G. p. gambiensis (95% confidence intervals as vertical bars). [file 1756-3305-4-18-S2.DOC]
